# Polyoxometalate/Cellulose Nanofibrils Aerogels for Highly Efficient Oxidative Desulfurization

**DOI:** 10.3390/molecules27092782

**Published:** 2022-04-27

**Authors:** Rui Song, Xueqin Zhang, Huihui Wang, Chuanfu Liu

**Affiliations:** 1 State Key Laboratory of Pulp and Paper Engineering, South China University of Technology, Guangzhou 510006, China; SRsongrui711@163.com (R.S.); whh@scut.edu.cn (H.W.); 2 College of Light Industry and Food Science, Zhongkai University of Agriculture and Engineering, Guangzhou 510225, China; zhangxueqin0228@163.com

**Keywords:** cellulose nanofibrils, phosphotungstic acid, oxidation desulfurization, catalyst

## Abstract

Polyoxometalate (POM) presents great potential in oxidative desulfurization (ODS) reaction. However, the high dissolubility of POM in common solvents makes it difficult to recycle. Besides, the small specific surface area of POM also limits the interaction between them and the substrate. Depositing polyoxometalates onto three-dimensional (3D) network structured materials could largely expand the application of POM. Here, the surfaces of cellulose nanofibrils (CNFs) were modified with very few (3-Aminopropyl) trimethoxysilane (APTS) to endow positive charges on the surfaces of CNFs, and then phosphotungstic acid (PTA) was loaded to obtain the aerogel A-CNF/PTA as the ODS catalyst. FT-IR indicated the successful deposition of PTA onto aminosilane modified CNF surfaces. UV-VIS further suggested the stability of PTA in the aerogels. BET and SEM results suggested the increased specific surface area and the relatively uniform 3D network structure of the prepared aerogels. TGA analysis indicated that the thermal stability of the aerogel A-CNF/PTA50% was a little higher than that of the pure CNF aerogel. Most importantly, the aerogel A-CNF/PTA50% showed good catalytic performance for ODS. Catalysis results showed that the substrate conversion rate of the aerogel A-CNF/PTA50% reached 100% within 120 min at room temperature. Even after five cycles, the substrate conversion rate of the aerogel A-CNF/PTA50% still reached 91.2% during the dynamic catalytic process. This work provides a scalable and facile way to stably deposit POM onto 3D structured materials.

## 1. Introduction

In recent years, desulfurization of fuel has been one of the most important ways to solve environmental problems. The common desulfurization methods in industry include hydrodesulfurization (HDS) [1,2,3], oxidation desulfurization (ODS) [4,5,6,7], biological desulfurization (BDS) [8,9], extraction desulfurization (EDS) [10,11] and adsorption desulfurization (ADS) [12,13,14]. Compared with the other technologies, ODS technology has many advantages, such as simple process, high desulfurization rate, mild conditions, convenient operation, and less pollution [4,5,6,7].

POM clusters have stable chemical properties, adjustable acidity and nanometer size, and are often used for catalytic ODS of fuel oil [15]. Discrete POM clusters can be viewed as transferable building blocks for the development of functional materials [16]. For example, organic–inorganic POM-containing hybrids or composites via non-covalent interactions (electrostatic static interaction, hydrogen bonding, van der Waals interactions) have been fabricated [17,18]. Compared with other ionic compounds, POM clusters possess lower surface charge density, which results in high dissolubility in a variety of solvents [19]. This is key for fabricating self-assembled POM-based hybrids or composites. Lay-by-lay (LbL) or Langmuir–Blodgett (LB) films obtained via the cation exchange method driven by electrostatic interaction are typical examples of POM-based self-assembled POM-containing materials [20,21]. Usually, in both POM-containing LbL and LB films, the thickness of the inorganic layer roughly corresponds to the thickness of the POM clusters. What is more, the dielectric constant of solvent, charge density, and solution pH all could tune and affect the structures (single-layered, hollowed, spherical, black-berry) of POM materials [22]. While many research works focused on the fabrication of POM-based zero to two-dimensional materials (including films, fibers, tunes, and belts, vesicles, and so on) [23], few works have reported on the nanostructure of POM-based three-dimensional (3D) materials [24,25]. 

Three-dimensional network structured material with high specific surface area and abundant reactive sites exhibit remarkable electric, mechanical, and catalysis properties. Loading POM onto 3D network structured materials could further enhance the interaction between catalyst and substrate, for example, by fixing POM onto the surface of resin through covalent bonds, and the results showed that the POM-organic skeleton solid catalyst possessed high selectivity. The catalyst could be reused many times and still have a good catalytic effect. However, the catalytic efficiency needs to be further improved and the catalytic conditions were not mild [26]. Silica was used as a fixation and an embedding medium for phosphomolybdic (PMo_12_) and phosphotungstic acid (PTA) to remove methyl phenyl sulfide [27]. The results showed that POM-doped silica aerogel possessed a high catalytic rate (4 h complete catalysis) and high selectivity. However, the preparation of the silica-aerogel based catalyst is complex and it needs to consume abundant chemical reagents. 

Cellulose nanofibrils (CNFs), originating from forestry biomass, could form 3D porous material through an easy freeze-drying process; they have attracted considerable attention in the fields of medicine, colloids materials, and bio-foams. Besides, there were abundant hydrophilic groups on CNFs, which also made CNFs a potential polymeric substrate to deposit POM. For examples, CNFs were grafted with a poly (ionic liquid), and the counterions of the poly (ionic liquid) were exchanged into POM anion clusters ([Co(OH_6_)Mo_6_O_18_]^3−^) [28]. In another work, researchers utilized CNFs to load [β-SiMo_3_W_9_O_40_]^n^^−^ by calcination at 400 °C. The removal efficiency of dibenzothiophene, benzohtiophene, and 4.6-dimethyldibenzothiophene could reach 88%, 89%, and 100%. While these materials presented good catalytic performance, the preparation process was complex and expensive, and could not take advantage of CNFs. In this work, we directly used CNFs as a polymer skeleton without calcination to immobilize Keggin type metal oxide clusters (phosphotungstic acid H_3_[P(W_3_O_10_)_4_]·nH_2_O, denoted as PTA) and fabricated 3D network structured aerogels. The surfaces of CNFs were cationized by APTS, which facilitates the deposition of PTA. The obtained CNF aerogels present excellent catalysis performance. The 3D network structured aerogel A-CNF/PTA presented good stability, recyclability, and scalability.

## 2. Results

### 2.1. CNF Aerogel Preparation

**Scheme 1:**
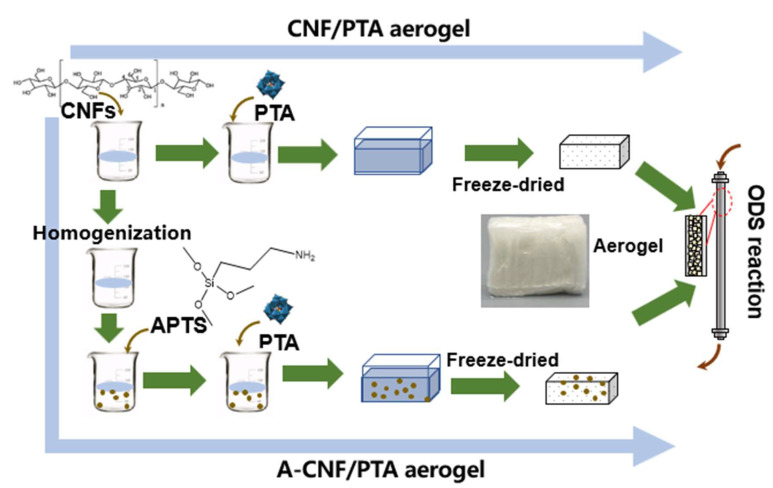
CNF aerogel preparation process.

**Figure 1:**
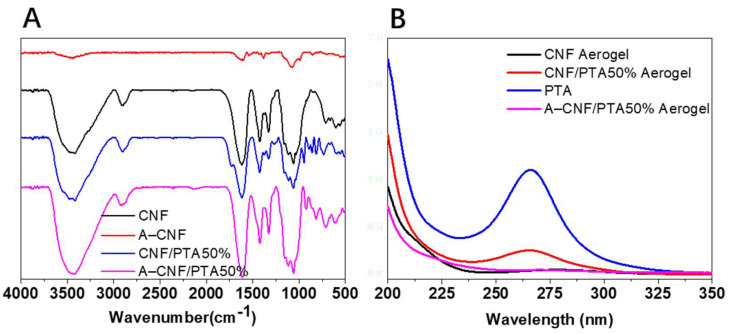
FT-IR spectra of the aerogels CNF, CNF/PTA50%, and A-CNF/PTA50% (**A**), and UV spectrum of the solution after immersion of aerogels (**B**).

The preparation process of the CNF aerogels is presented in Scheme 1. PTA was added into CNF suspension before and after aminosilane modification. The obtained mixture was stirred and freeze-dried to obtain the CNF aerogels CNF/PTA and A-CNF/PTA. The chemical structures of the CNF aerogels were characterized with FT-IR. As shown in Figure 1, the peak at 1536 cm^−1^ is attributed to -NH_2_ [27], and the peaks at 1086 and 1066 cm^−1^ correspond to the stretching vibration of Si-O-C [27]. These peaks appeared in the FT-IR spectra of samples A-CNF and A-CNF/PTA50%, suggesting a successful reaction between CNFs and APTS. Besides, the peaks at 790 and 450 cm^−1^ corresponding to the symmetric stretching vibration and bending vibration of Si–O–Si could also be observed in the FT-IR spectrum of A-CNF/PTA50% [29]. These further confirmed the occurrence of a reaction between CNFs and APTS. The peaks at 1080, 978, 885, 805, and 523 cm^−1^ corresponded to the bending vibrations of different oxygen atoms (P-O_a_, W=O_d_, W-O_b_-W, W-O_c_-W, W-O-P) from PTA (Figure S2), which could be clearly observed in the FT-IR spectra of the aerogels CNF/PTA50% and A-CNF/PTA50% [29,30,31,32,33,34]. This indicated that PTA was successfully loaded on unmodified and modified CNFs. 

The interaction between PTA and surface modified CNFs was obviously enhanced, which could be confirmed by the dissolution behaviors of the CNF aerogels. As shown in Figure 1B, when the aerogel CNF/PTA50% was soaked into acetonitrile, PTA partially dissolved into the solvent, which resulted in the obvious adsorption peak in the UV spectra. However, the UV spectrum of the aerogel A-CNF/PTA50% almost showed no presence of PTA adsorption peaks after depositing PTA onto aminosilanized CNFs [35]. This proved the CNF surface aminosilane modification could significantly enhance the stability of PTA on the aerogels [36].

### 2.2. Morphology and Porosity of CNF Aerogels

**Figure 2:**
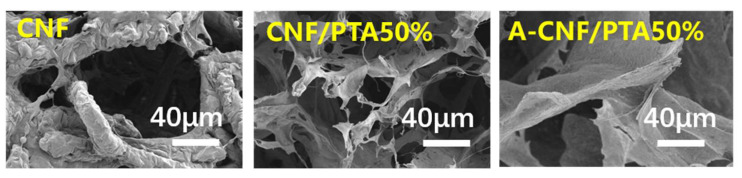
SEM images of the aerogels CNF, CNF/PTA50%, and A-CNF/PTA50%.

The micromorphology of the aerogels was characterized by SEM [37,38]. As shown in Figure 2, compared with the SEM image of the pure CNF aerogel, the aerogels CNF/PTA50% and A-CNF/PTA50% showed the more uniform 3D network structures. This would benefit for the interaction between the substrate and the PTA, resulting in the improved catalysis efficiency [26,39,40,41,42]. The formation of the relatively uniform 3D networks is highly related with the hydrogen bonding interaction between PTA and CNFs. Compared with the aerogel CNF/PTA50%, the surface of the aerogel A-CNF/PTA50% became rougher, which is highly relevant to the enhanced electrostatic interaction between the positively charged aminosilanized CNFs and the anionic clusters of PTA.

**Figure 3:**
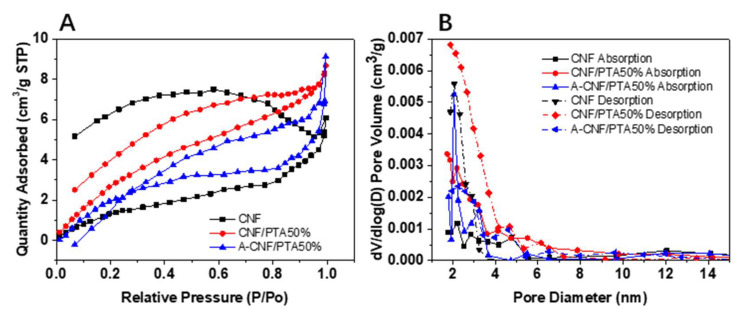
Adsorption and desorption curves of the aerogels CNF, CNF/PTA50%, and A-CNF/PTA50% (**A**), pore size distribution curve of aerogels obtained by BJH method (**B**).

The porosity of CNF aerogels was explored by BET. As shown in Figure 3A, compared with the pure CNF aerogel, the specific surface area of the aerogels CNF/PTA50% and A-CNF/PTA50% increased to 14.71 and 7.08 m^2^/g, respectively (Table S1). Besides, compared with the pure CNF aerogel, the average pore size diameter of the aerogels CNF/PTA50% and A-CNF/PTA50% both decreased to 4.36 and 6.15 nm (Figure 3B), respectively. The increased specific surface area and decreased pore diameter both facilitate the interaction between substrate and catalyst, resulting in improved catalysis efficiency [43,44,45]. 

### 2.3. Static Catalysis

PTA shows high catalytic activity for ODS reaction. In this paper, methyl phenyl sulfide was used as the substrate for ODS reaction, transforming into the middle product methyl phenyl sulfone (MPSX), and then transforming the final catalytic product methyl phenyl sulfone (MPSO) under the catalytic oxidation of CNF/PTA aerogels [27,46,47,48,49]. Methyl phenyl sulfide is toxic, but sulfoxide and sulphone are widely used in many fields, such as pharmaceutical material chemistry [50].

**Figure 4:**
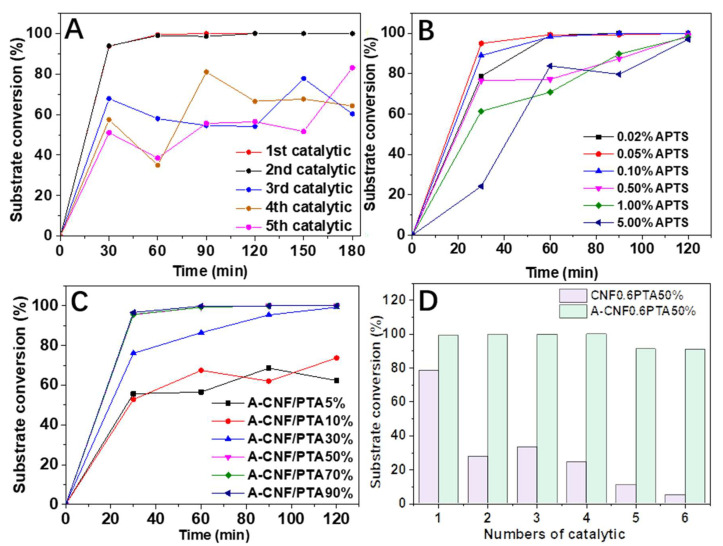
Cyclic catalytic performance of the aerogel CNF/PTA50% (**A**), catalytic performance of aminosilanized CNF aerogels (**B**), catalytic performance of A-CNF/PTA aerogels with different PTA content (**C**), cyclic catalytic performance contrast between the aerogels CNF/PTA50% and A-CNF/PTA50% (**D**).

The substrate could not be absorbed by the CNF aerogels, while the catalytic products could be absorbed by the CNF aerogels, as evidenced by the GC spectra (Figure S5). This means that the disappearance of substrate peaks in the GC spectrum is not due to adsorption by aerogels, but transformation, and it also indicates that aerogels can collect part of intermediates and products. Therefore, in this paper, the catalysis efficiency was evaluated by the substrate conversion rate, which was calculated according to Equations (1) and (2). The substrate conversion rate of the aerogels CNF/PTA50% reached nearly 100% within 60 min at the first-time catalysis. However, the substrate conversion rate of the aerogel CNF/PTA50% started to decrease from the third-time catalysis, and sharply decreased to 60% at the fifth-time catalysis (Figure 4A). This is because that part of the PTA fell off from the CNF aerogel during the catalysis, resulting in reduced catalytic efficiency [25,51,52,53,54,55]. This was consistent with the dissolution behavior of the CNF/PTA aerogels. 

Loading PTA onto the surfaces of aminosilanized CNFs can effectively prevent PTA falling off from the aerogels. The positive charges on aminosilanized CNF surfaces benefit for PTA stabilization, while excessive aminosilane groups suppressed the catalysis activity of PTA, as shown in Figure 4B. This is due to the aminosilane groups being slightly alkaline in water, which suppresses the catalytic activity of PTA. The excessive aminosilane groups would also affect the stability of PTA [56]. However, CNFs with slight APTS (0.1%) modification still showed the poor catalytic stability during the cycle catalysis process (Figure S3), in which the substrate conversion rate reduced to about 60% at the fourth-time catalysis. However, the CNF aerogel with 1.0% APTS modification showed a good substrate conversion rate (82.64%) at the fourth-time catalysis (Figure S3). Compared with the initial catalysis, the substrate conversion rate of the aerogel A-CNF/PTA50% slightly decreased, which probably resulted from the poor dispersibility of CNFs and the aggregated structure resulting in the destruction of the aerogels. When the dosage of APTS was fixed at 1.0% and the dosage of PTA was higher than 50%, the substrate conversion efficiency of the aerogel A-CNF/PTA50% could reach 100% within 30 min (Figure 4C). To improve the stability of PTA on the CNF surfaces and preserve the high catalytic performance of PTA, the optimal preparation conditions for CNF aerogels were as follows: Volume ratio 1.0% (APTS/CNF suspension, V/V) of APTS, and mass percentage 50% (PTA/CNF, m/m) of PTA.

### 2.4. Dynamic Catalysis 

**Figure 5:**
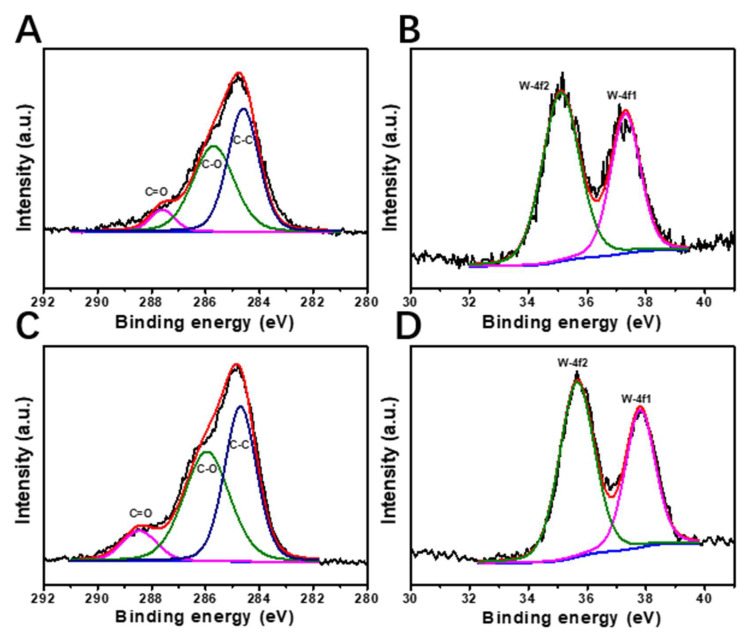
C1s spectra of A-CNF/PTA50% before (**A**) and after (**C**) catalysis. W4f spectra of A-CNF/PTA50% before (**B**) and after (**D**) catalysis.

In fact, compared with static catalysis experiments, dynamic catalysis shows greater potential for industrial applications [57]. Static catalysis experiments have confirmed the optimal substrate conversion rate of the aerogel A-CNF/PTA50% under mild catalytic conditions. Unfortunately, we found that a small amount of CNFs fell off from the aerogels during the catalytic process, which might have resulted from the highly aggregated and poorly dispersed structure of CNFs. In order to avoid the destruction of the CNF aerogel affecting the catalytic process, we further homogenized the raw material and diluted it to 0.6 wt% before modifying it with APTS. Figure 5D shows the comparison of cyclic substrate efficiency of the aerogels CNF/PTA50% and A-CNF/PTA50%. As shown in Figure 4D, the aerogels CNF0.6/PTA50% and A-CNF0.6/PTA50% showed highly different dynamic catalysis behavior. The substrate conversion rate of the aerogel A-CNF0.6/PTA50% was up to 90% even after five cycles, while the substrate conversion rate of the aerogel CNF0.6/PTA50% was lower than 50% after five cycles. This further confirmed that depositing PTA onto positively charged CNFs could preserve the high catalysis performance of PTA and avoid the dissolution of PTA. 

### 2.5. Changes of CNF Aerogels before and after Catalysis

In order to investigate the elemental composition change, the aerogel A-CNF/PTA50% before and after catalysis were characterized with XPS [30]. As shown in Table 1 and Figure 5, the peaks at 284.6 eV, 285.7 eV, 287.6 eV are attributed to C-C, C-O, C=O, and the binding energy at 37.3 and 35.1 eV correspond to W-4f1 and W-4f2, respectively [26,30,31,34]. The content of C=O and C-O increased from 6.98% to 8.36% and 44.45% to 46.46%, respectively, while the content of C-C decreased from 48.37% to 45.18%. This is probably due to the oxidation reaction on CNFs during the catalytic process, in which H_2_O_2_ partially oxidized CNFs resulting in the increased C=O and C-O [58,59]. The content of the different valence states of element W basically remained unchanged, indicating a stable chemical structure of PTA during the catalytic process.

### 2.6. Thermal Stability of CNF Aerogels

**Figure 6:**
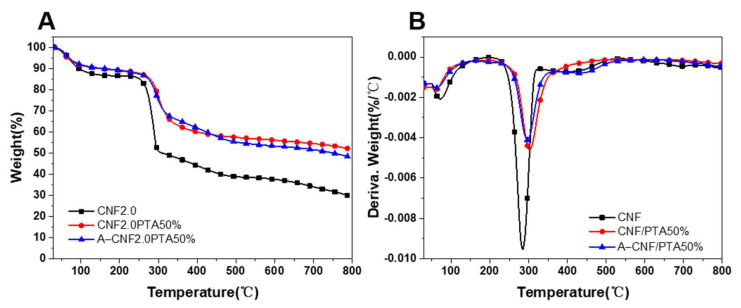
Thermogravimetric analysis (**A**) and derivative thermogravimetric analysis (**B**) curves of the CNF aerogels.

The thermal stability of the CNF aerogels was assessed by thermogravimetric analysis (TGA) [20]. As shown in Figure 6, the slight weight loss lower than 100 °C was due to loss of water that is possibly from the adsorbed moisture of the aerogels during storage. The main pyrolysis process of cellulose is accompanied by a large weight loss [60]. Compared with the CNF aerogel, the initial degradation and mid-degradation temperatures of the aerogel A-CNF/PTA50% increased from 284.2 °C to 293.2 °C, and from 291.5 °C to 295.8 °C (increased by 3.2% and 0.9%, respectively). Correspondingly, the temperature at the maximum degradation rate of the aerogels CNF/PTA50% and A-CNF/PTA50% decreased by 16.3 °C and 17.7 °C, respectively (Figure 6B). It can be seen that with the addition of PTA, the thermal stability of the CNF aerogels is slightly improved. The aerogel A-CNF/PTA showed the slightly enhanced thermal stability, which can ensure the smooth catalytic process without degradation of catalyst [61].

## 3. Materials and Methods

### 3.1. Materials

CNF powder was directly purchased from Qihong Tech. Co. (Guilin, China). Phosphotungstic acid (H_3_O_40_PW_12_·xH_2_O, PTA), (3-Aminopropyl) trimethoxysilane (APTS), and methyl phenyl sulfide (AR) with analytical grade were obtained from Macklin Co. (Shanghai, China). Octane (chromatographic grade), *p*-tolysulfide, *p*-tolysufoxide, *p*-tolysulfone, and acetonitrile with chromatographic grade were purchased from Aladdin Co. (Shanghai, China). Hydrogen peroxide (H_2_O_2_) was obtained from Guangzhou Chemical Co. (Guangzhou, China).

### 3.2. CNF Aerogel Preparation

The CNF powder was redispersed into DI water by ultrasonic treatment. PTA (PTA: CNF, m: m, 10%, 30%, 50%, 70%, 90%) was added into 20 g of CNF suspension with concentration 2.0 wt%. The mixture was stirred at room temperature (25 °C) for 30 min, and then the mixture was frozen in an ultra-low temperature refrigerator (−40 °C). Then the frozen CNF/PTA samples were immediately transferred into a freeze dryer (FM25XL-70, SP Scientific) for 48 h to obtain the aerogels CNF/PTA.

APTS with different dosage (APTS: CNF suspension, V:V, 0.02%, 0.05%, 0.1%, 0.5%, 1.0%, 5.0%) was added into 20 g of CNF suspension with concentration 2.0 wt%. The mixture was stirred continuously at room temperature (25 °C) for 2 h to obtain CNFs modified by APTS (A-CNFs). Then a different amount of PTA (PTA: CNFs, m:m, 10%, 30%, 50%, 70%, 90%) was added into 20 g of A-CNF suspension and stirred at room temperature (25 °C) for 30 min. The obtained mixture was frozen in the ultra-low temperature refrigerator (−40 °C), and then freeze dried for 48 h to obtain the aerogels A-CNF/PTA.

CNFs was further homogenized and dispersed into 0.6 wt% suspension; 1.0% APTS was added into 20 g of 0.6 wt% CNF suspension. The mixture was stirred at room temperature for 2 h and then 50% PTA was added into the suspension and stirred at room temperature (25 °C) for 30 min. After pre-freezing in an ultra-low temperature refrigerator and freeze-drying for 48 h, A-CNF0.6/PTA50% was obtained.

### 3.3. Characterization

The samples were characterized by FT-IR (Nicolette IS50-Nicolet Continuum, Thermo Fisher Scientific, Waltham, MA, USA), and the spectra were recorded in 4000–400 cm^−1^. The microstructures of aerogels were obtained by SEM (Sigma 300, Zeiss, Jena, German). The specific surface area was analyzed with BET (ASAP 2020, Micromeritics Company, Lawrenceville, GA, USA). The thermal stability of CNF aerogels was measured by TG-DSC (TGA, Mettler TGA/DSC3+, Columbus, OH, USA). The CNF aerogels before and after catalysis were analyzed by XPS (Axis Ultra DLD, Kratos, Manchester, UK). The dissolution of the catalysts was measured by using ultraviolet-visible spectrophotometry (UV-VIS) (UV-1800, Shimadzu, Kyoto, Japan). Gas chromatograph (GC) (GC7920-TF2A, Zhong Jiao Jin Yuan, Beijing, China) was used to detect the conversion rate of the substrate during the catalyst process.

### 3.4. Dissolution Behaviors of the CNF Aerogels

The 7 mg aerogel samples were immersed in a 6.6 mL solution of acetonitrile and H_2_O (acetonitrile: H_2_O, V:V, 10:1), respectively. After standing for 48 h, the solution was analyzed by UV-VIS.

### 3.5. Static Catalytic Experiments

The catalytic experiments of the CNF aerogels were performed as follows. At different stages of the catalytic reaction, 10 μL of the substrate solution (260 μL substrate, 130 μL n-octane, 600 μL H_2_O_2_ and 6 mL acetonitrile and 7 mg catalyst) was collected and injected into the gas chromatograph. Catalytic experiments were carried out at room temperature (25 °C) and samples were taken every 30 min. The whole catalytic reaction process was 90 min. The substrate conversion rate can be calculated according to the change of relative value of peak area of substrate and internal standard. The substrate conversion rate was calculated according to the following equation:

(1) C=I(species,t)I(octane,t)I(sulfide,t0)I(octane,t0)

(2) $$\alpha_{t}=\frac{C_{0}-C_{t}}{C_{0}}\times 100\%$$

in which *I* (*species*, *t*) is the peak area of the sample at time *t*; *I* (*octane*, *t*) is the peak area of the internal standard n-octane at time *t*; *I* (*sulfide*, *t*_0_) is the peak area of the substrate at time *t*_0_; *I* (*octane*, *t*_0_) is the peak area of the internal standard n-octane at time *t*_0_; *C* is the relative value of the peak; *α_t_* is the substrate conversion rate.

### 3.6. Dynamic Catalytic Experiments

A 28 mg amount of the aerogel A-CNF/PTA was filled into the catalytic column. The substrate solution (the same as in Section 3.4) flowed through the catalytic column at a rate of 2 mL/h. The whole process was carried out at 40 °C and lasted for 2 h. A 10 μL amount of the liquid which flowed out of the catalytic column was collected and injected into the gas chromatograph. The substrate conversion rate was calculated according to Equations (1) and (2). 

## 4. Conclusions

In summary, we successfully prepared aminolisanized CNFs for depositing PTA (the aerogel A-CNF/PTA), and we proved that the aminosilanization of CNF surfaces could obviously enhance the stability of PTA. The optimal preparation conditions of the A-CNF/PTA aerogel were as follows: Volume ratio of APTS 1.0%, and mass percentage of PTA 50%. The aerogel A-CNF/PTA50% presented a relatively uniform 3D network structure with a specific surface area of 7.08 m^2^/g, and the average pore size was 6.15 nm. The aerogel presented high static catalytic activity; that is, the substrate conversion rate was close to 100% within 30 min at room temperature. The aerogel A-CNF/PTA50% also showed excellent dynamic catalytic performance during the cycle catalytic process, in which the substrate conversion rate was above 90% after catalysis occurred five times. The facile strategy developed here may offer great possibility for the production of economical and recyclable biomass-based ODS catalysts.

## Figures and Tables

**Table 1:** Elemental analysis of A-CNF/PTA50% before and after the catalytic process.

Sample	Elemental Quantitative Analysis by XPS (%)				
	C-C	C-O	C=O	W-4f1	W-4f2
**Before**	48.37	44.65	6.98	39.27	60.73
**After**	45.18	46.46	8.36	38.50	61.50

## Data Availability

Not available.

## Supplementary Materials

The following supporting information can be downloaded at: https://www.mdpi.com/article/10.3390/molecules27092782/s1, Table S1. BET surface areas, pore volumes and pore sizes of the aerogels; Table S2. BJH surface areas, pore sizes of the aerogels; Figure S1. Catalysis process of PTA; Figure S2. The structure of PTA (H3O40PW12·xH2O); Figure S3. Effect of different APS addition amount on cyclic catalysis; Figure S4. UV spectrum of PTA (H3O40PW12·xH2O); Figure S5. GC chromatograms of different catalytic reactions at 0 min and 90 min; Figure S6. SEM of aerogels before (A, B) and after catalytic reaction (C, D).

## Sample Availability

Samples of the compounds are not available from the authors.
