# Supplementary material for: Polyoxometalate/Cellulose Nanofibrils Aerogels for Highly Efficient Oxidative Desulfurization
Source: Molecules. 2022 Apr 27;27(9):2782. doi: 10.3390/molecules27092782 (PMC9101072; doi:10.3390/molecules27092782)
Supplement: Supplementary file 1 [file molecules-27-02782-s001.zip › molecules-1675890-supplementary.pdf]

Polyoxometalate/cellulose nanofibrils aerogels for highly efficient oxidative desulfurization

Rui Song [1], Xueqin Zhang [2], Huihui Wang [1], and Chuanfu Liu [1,*]

1 State Key Laboratory of Pulp and Paper Engineering, South China University of Technology, Guangzhou China, SRsongrui711@163.com (R.S); zhangxueqin0228@163.com (X.Z.); whh@scut.edu.cn (H.W.).
2 College of Light industry and Food Science, Zhongkai University of Agriculture and Engineering, Guangzhou, Guangdong, 510225, China
* Correspondence: chfliu@scut.edu.cn (C.L.); Tel.: +86-20-8711-1735 (C.L.)

Table S1. BET surface areas, pore volumes and pore sizes of the aerogels.

Sample	BET		
	BET surface area (m^2/g)	Average pore size (nm)	Total pore volume (cm^3/g)
**CNF**	6.77	5.56	0.01
**CNF/PTA50%**	14.71	3.65	0.01
**A-CNF/PTA50%**	7.08	7.97	0.01

Table S2. BJH surface areas, pore sizes of the aerogels.

Sample	BJH of pores			
	Adsorption cumulative surface area (m^2/g)	Desorption cumulative surface area (m^2/g)	Adsorption average pore diameter (nm)	Desorption average pore diameter (nm)
**CNF**	4.27	8.50	6.90	4.85
**CNF/PTA50%**	10.22	17.80	4.36	2.97
**A-CNF/PTA50%**	6.04	7.41	6.15	4.24

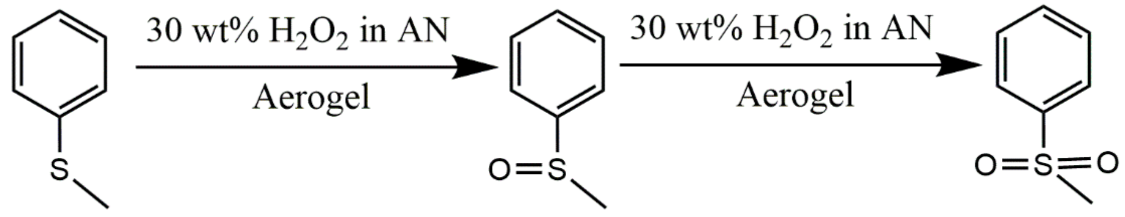

Figure S1. Catalysis process of PTA.

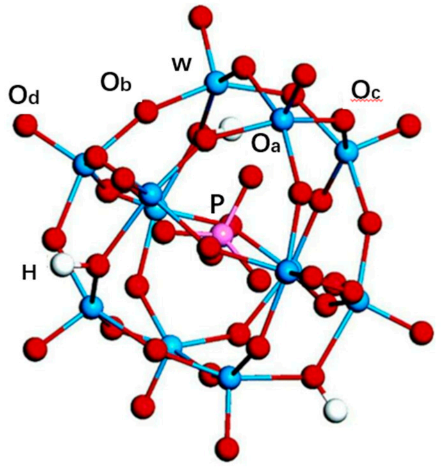

Figure S2. The structure of PTA ($H_3O_{40}PW_{12}·xH_2O$).

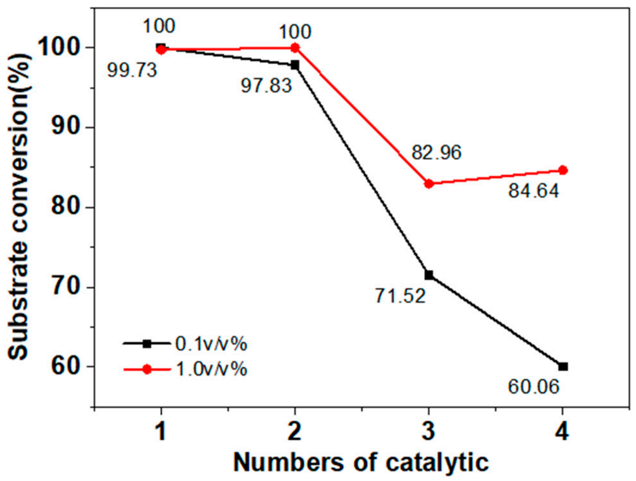

Figure S3. Effect of different APS addition amount on cyclic catalysis.

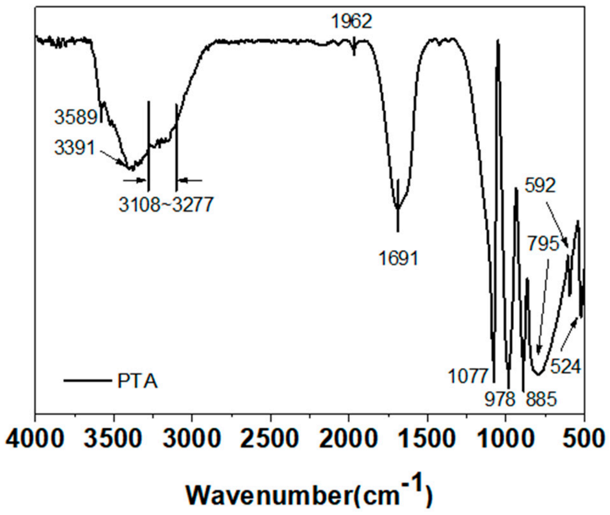

Figure S4. UV spectrum of PTA ($H_3O_{40}PW_{12}{\cdot}xH_2O$).

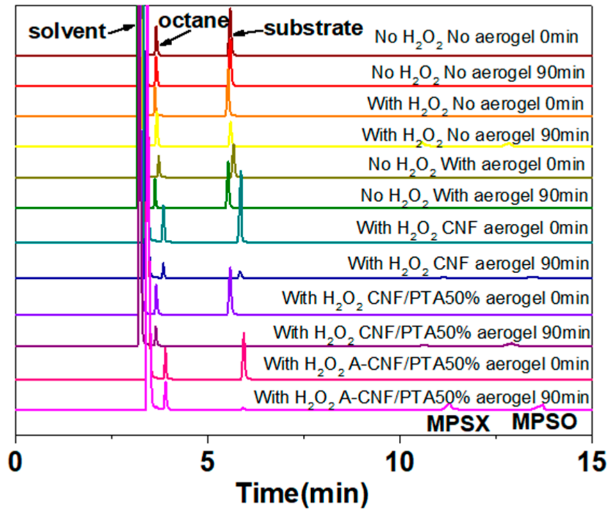

Figure S5. GC chromatograms of different catalytic reactions at 0min and 90min.

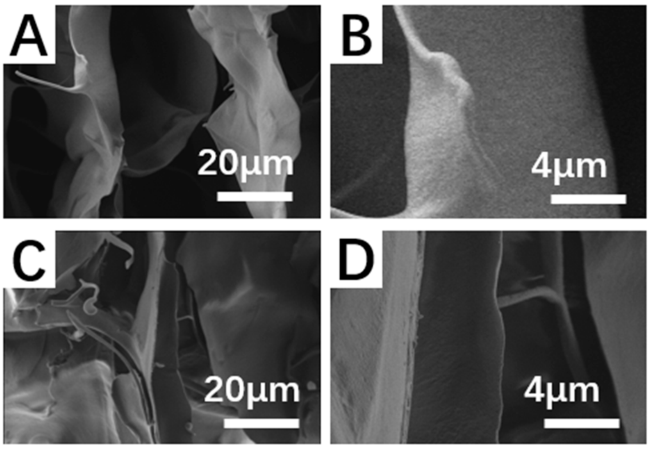

Figure S6. SEM of aerogels before(A,B) and after catalytic reaction(C,D).
